# Diagnostic performance of ultra-low field neurological magnetic resonance imaging: a systematic review protocol

**DOI:** 10.1136/bmjopen-2026-118511

**Published:** 2026-09-04

**Authors:** Paul Lockwood, Elizabeth Barrett

**Affiliations:** 1 School of Nursing, Midwifery, Allied and Public Health, Canterbury Christ Church University, Canterbury, Kent, UK; 2 Radiology, Dartford and Gravesham NHS Trust, Dartford, UK

**Keywords:** Magnetic Resonance Imaging, Neuroradiology, Head & neck imaging

## Abstract

**Introduction:**

The aim of this systematic review protocol is to provide the current reported level of diagnostic performance of ultra-low field MRI (ULF-MRI) in brain imaging of both paediatrics (including neonates and infants) and adults from a range of neurological conditions.

**Methods and analysis:**

We will use the population/problem, intervention, comparison and outcome framework to break down the keywords to search PubMed, Medline, CINHAL, Embase, Web of Science, Scopus, Cochrane Central Register of Controlled Trials and Google Scholar without restriction of geographical location, study design and publication status. Other sources such as the reference list of selected studies will also be searched, and the search will be documented with a Preferred Reporting Items for Systematic Reviews and Meta-Analyses (PRISMA) flow chart. The Quality Assessment of Diagnostic Accuracy Studies 3 tool will be used to assess the quality of the retrieved studies. After screening the studies, a meta-analysis for the primary outcome of individual and pooled diagnostic accuracy will be conducted using Meta-DiSc V1.4. Results expressed as forest plots, pooled heterogeneity values, 95% CIs, probability values and summary receiver operating characteristic curve plots will be used for data synthesis. The review commenced in January 2026 and is anticipated to conclude in June 2026. At the time of protocol submission, study identification, screening, data extraction, quality assessment and evidence synthesis have not yet commenced.

**Ethics and dissemination:**

The results of this systematic review will be disseminated in a peer-reviewed journal and presented at a relevant conference. Data do not include animal or human participant recruitment or personal individual data. All data are already published in the public domain; therefore, ethical approval is not required.

**PROSPERO registration number:**

PROSPERO 1308164

**STRENGTHS AND LIMITATIONS OF THIS STUDY**

- The systematic review will adopt a comprehensive and objective evaluation of the diagnostic performance of ultra-low field MRI (ULF-MRI) in brain imaging for a range of common neurological conditions.
- Owing to the potential for heterogeneity arising from the variety of neurological conditions imaged in these studies, results will be reported as overall diagnostic accuracy.
- Two researchers will independently conduct study selection, data extraction and quality assessment to minimise bias and ensure comprehensive inclusion of relevant studies.
- A key strength is the inclusion of diverse clinical settings, environments, patient populations and comparisons to control imaging; this broad inclusion will enhance the external validity by demonstrating the potential applicability of ULF-MRI across a range of real-world healthcare contexts.

## Introduction

 MRI is a standard non-invasive diagnostic imaging examination for many anatomical and functional structures of the human body. Image quality is often determined by the magnetic field strength measured in Tesla (T) or milliTesla (mT) at the centre of the magnet and determines the frequency of the electromagnetic radio frequency (RF) energy pulses to collect the signal to create an image.^1^

**Figure 1:**
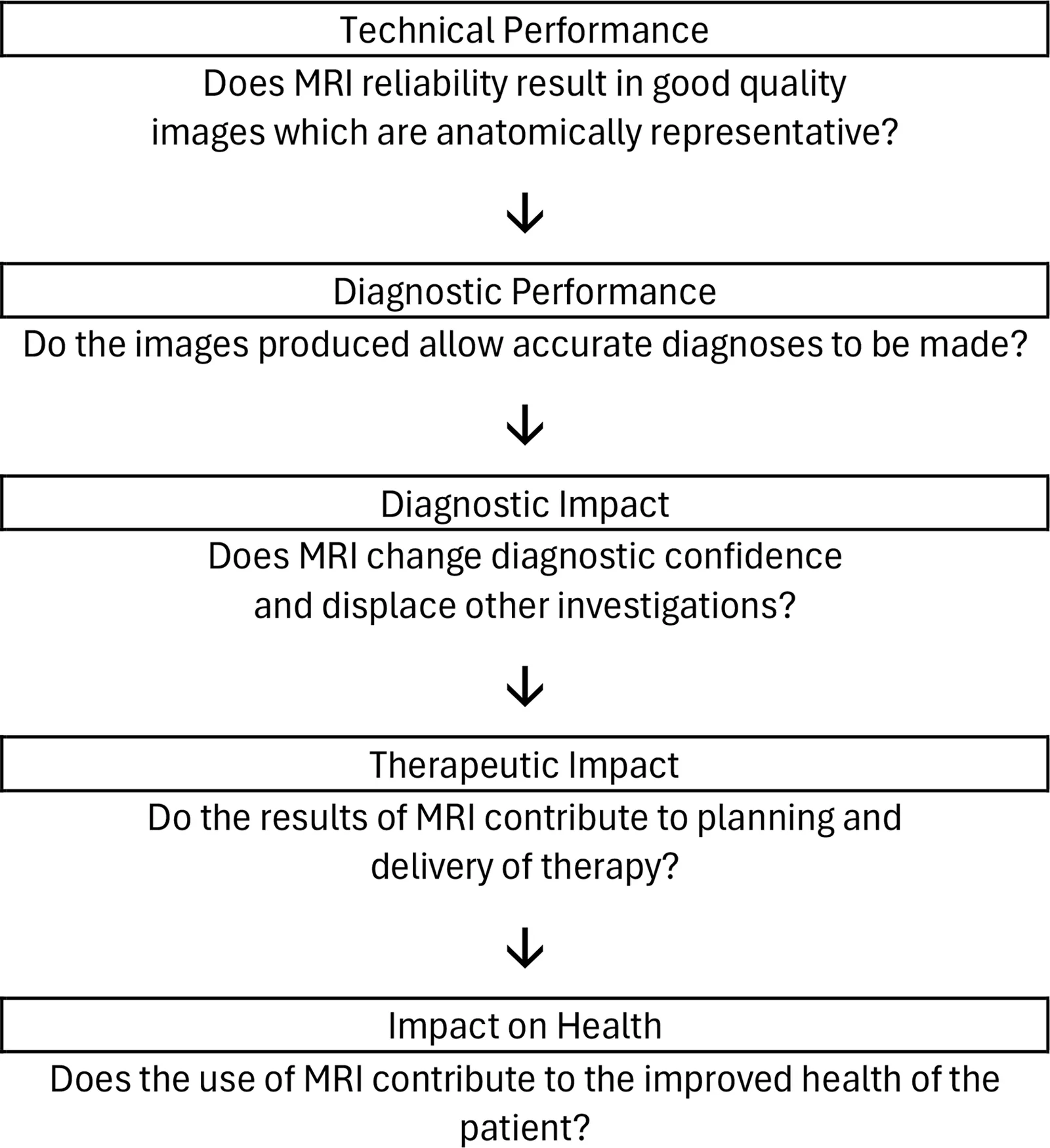
Mackenzie and Dixon^13^ five-stage hierarchical framework to assess the effects of MRI.

Ultra-low field MRI (ULF-MRI) is a non-invasive and non-ionising diagnostic imaging scanner with a magnetic field strength of <0.1T, or <100mT; an example would include the portable hyperfine scanner with a field strength of 0.064T. As an imaging modality, ULF-MRI is becoming popular owing to its point-of-care design and low magnetic field strength, which allows it to be used outside a normal clinical environment (hospital) to serve hard-to-reach communities for instant imaging without the high-cost specialist room requirements. To incorporate new and emerging imaging technologies into mainstream imaging practices, it is essential to have a robust body of evidence. In diagnostic imaging, established hierarchical frameworks^2^ to evaluate the clinical efficacy of imaging modalities have existed since the 1970s. Specifically, for MRI, the Mackenzie and Dixon^3^ hierarchical framework is a five-stage framework^3^ (figure 1) that differentiates each inherent step of MRI performance from technical to diagnostic, to the influence on patients’ diagnosis, management and health.

Studies have already been published on the level 1 technical efficacy^2^ of the early development,^4^ testing and optimisation^5^ of ULF-MRI for neurological imaging. These studies have mainly focused on assessing datasets of imaging to define outcome metrics around image quality, such as signal-to-noise ratio, contrast-to-noise, spatial resolution (often including anatomical boundary differentiation,^6^ such as grey–white matter segmentation^7^ or ventricular boundaries), uniformity, image sharpness and acceptability and artefact severity. They have also assessed the reproducibility of ULF-MRI in accurately replicating high-field (HF) MRI (1.5–3T) brain volumetric assessments^8^ (anatomical integrity and anatomical structural difference measurements for clinical benchmarking), as well as Dice similarity coefficients for segmentation overlap, with the level of agreement between ULF-MRI and HF-MRI in structural morphometry. Volumetric accuracy is an indication of the technical performance and reliability of the ULF-MRI system to measure either the whole brain volume, grey/white matter volume,^9^ hippocampal volume,^9^ cerebrospinal fluid volume^9^ or ventricular volume.

This systematic literature review protocol aims to assess the current published level 2^2^ evidence on the diagnostic accuracy to establish a baseline performance for neurological conditions and pathologies with ULF-MRI. The review will include areas such as stroke,^5^ hydrocephalus,^10^ intracranial pathology,^11^ multiple sclerosis,^12^ neurodegenerative disorders^13^ and other neurological conditions in which ULF-MRI has been applied.

For acceptance into the clinical pathway, the evidence will need to demonstrate that the diagnostic accuracy of ULF-MRI is equivalent to, or within a defined acceptable clinical tolerance margin and not lower than, that of HF-MRI (non-inferiority). The study will not assess the higher Fryback and Thornbury^2^ clinical efficacy of downstream outcomes on the treatment and management of patients or the benefits to clinical service delivery.

## Methods and analysis

### Inclusion criteria for study selection

#### Patient and public involvement

A small focus group of Patient and Public Involvement and Engagement (PPIE) volunteers from the university^14 15^ were engaged^16–18^ in the project during the brainstorming and design stages to provide input on the topic, theme, outcome measurements to be used^19 20^ and the relevance of the topic to their experience^21^ (service users) of improving healthcare access (neurological imaging in non-traditional settings such as the portability of ULF-MRI for environments such as intensive care units where patients cannot be moved or community settings where patients live in remote, rural or under-resourced areas). Recruitment to the focus group was through a centrally managed PPIE coordinator, with the focus group conducted online to reduce the burden of participant costs and accessibility issues. Questions did not address the burden of the intervention for the study participants or the time required for study participation, as this is a systematic literature review study with no human participants involved in the study design. In accordance with good research practice guidelines, a summary with a link to the full final results paper will be disseminated to the PPIE group at the end of the study.

### Types of studies

This review will include diagnostic accuracy studies; randomised controlled trials (RCTs); non-randomised comparative studies if there is not enough evidence from the RCTs; cohort studies; case–control studies; cross-sectional studies; case series and case reports (even if they report relevant safety or effectiveness data); reviews and meta-analyses; editorials and commentaries; conference abstracts; and in vitro (laboratory-based) studies.

### Types of participants

This review will apply the population/problem, intervention, comparison and outcome (PICO) framework^22^ (table 1) to develop the primary objective: in patients undergoing brain imaging for suspected neurological conditions/symptoms (P), how does ULF-MRI (I) compare with conventional HF-MRI and/or CT (C) in diagnostic accuracy for detecting intracranial abnormalities (O)?

**Table 1:** PICO breakdown and structure of the research question

Population/Problem (P)	Intervention (I)	Comparison (C)	Outcomes (O)
Patients (human) undergoing brain imaging for neurological conditions. (eg, brain tumours, stroke, traumatic brain injury, epilepsy and infection)	Ultra-low field neurological MRI (≤0.1–0.3 T)	Standard neurological imaging such as MRI brain scans (1.5 T/3T), CT head scans or clinical reference standard. (Depending on the primary comparator reported)	Diagnostic accuracy metrics reported

PICO, population/problem, intervention, comparison, and outcomes.

### Types of interventions

Studies reporting primary data that have been published and peer-reviewed will be included if they (1) involve human participants undergoing brain imaging, (2) use ULF-MRI (≤0.3T), (3) report diagnostic performance or image evaluation and (4) compare findings with conventional HF-MRI, CT or other appropriate clinical imaging reference standards. All studies will be considered regardless of the country of research, settings, study design, community of patients sampled or MRI manufacturer/vendor or MRI protocol used. No restriction on publication date will be applied.

Studies involving non-brain/head imaging (such as other anatomical areas), phantom studies (physics testing), animal imaging, lack of diagnostic outcome data or artificial intelligence (AI) tools as the reader/interpretation or AI testing to improve image quality, AI MRI sequences or AI MRI protocols will be excluded. Non-English-language published studies will be excluded because of the potential for translation errors that could introduce inaccuracies in the interpretation and analysis of findings.

All retrieved citations will be exported to reference management software. Duplicates will be removed, and the final list will be prepared for title and abstract screening (Preferred Reporting Items for Systematic Reviews and Meta-Analyses (PRISMA) flowchart). All search activity will be documented for final reporting.

### Types of outcome assessments

The primary outcomes include (but are not exhaustive, depending on outcome measures published in studies) human participants as readers of imaging (most commonly a consultant neuroradiologist). This study will not be using AI as a reader. Outcome metrics will include data in 2×2 contingency table results (true negative, true positive, false negative and false positive), sensitivity (true-positive rate), specificity (true-negative rate), accuracy (ability to detect pathology) and agreement (to a reference standard of the same imaging).^23^ With the addition of reviewing reporting positive-predictive values (PPV)/negative-predictive values (NPV) (probability of having/not having conditions given a positive/negative test result), probability values (p-values) of the test, likelihood ratios, summary receiver operating curve (SROC) and area under the curve (AUC).

Secondary outcomes include the sample size, lesion detection rate, image quality score, confidence levels, reference standards and environment of the imaging (community, laboratory, hospital, etc). Secondary outcomes will not record brain tissue measurements, subcortical grey/white matter structural volumes, etc.

### Search methods for identification of studies

#### Electronic searches

The literature search will be conducted between February and March 2026. Search strategies will be developed specifically for this systematic review, using a combination of controlled vocabulary (hierarchical controlled vocabularies for health sciences (eg, Medical Subject Headings) and free-text terms: adult, brain, brain disease, brain infarction, brain injury, brain haemorrhage, brain neoplasms, brain injuries, brain oedema, central nervous system, cerebrovascular trauma, cerebral haemorrhage, hydrocephalus, intracranial arterial diseases, dementia, stroke, humans, MRI, neuroimaging, neuroradiography, patient outcome assessment, point-of-care systems, sensitivity and specificity, systematic reviews as topic and workflow. As ULF-MRI is a new term, keywords added were ultra low field, ultra-low-field, low-field MRI, portable MRI, 0.064T, 64mT, 0.1T and 0.2T.

The Boolean terms include the following:

(“MRI OR “magnetic resonance imaging” OR “ultra low field” OR “ultra-low-field” OR “low-field MRI” OR “portable MRI” OR “0.064T” OR “64mT” OR “0.1T” OR “0.2T” OR “portable MRI” OR “portable scanner” OR “Low-cost MRI” OR “Low-field MRI” OR “low-field brain MRI” OR “Point of Care MRI” OR pMRI OR POC MRI OR Mobile MRI OR mMRI OR “Mobile point of care” MRI OR “Low Tesla Magnetic resonance imaging” OR “Low strength Magnetic resonance imaging” OR “Low strength” MRI OR “bedside MRI” OR “bedside portable MRI” OR “brain only MRI” OR “head only MRI” OR “Hyperfine Swoop” OR Hyperfine OR “Halbach MRI” Or Halbach”)

#### AND

(brain OR intracranial OR neurological OR stroke OR tumor OR tumour OR epilepsy OR haemorrhage OR hemorrhage OR TBI OR “Brain Diseases” OR brain OR neurological OR intracranial)

#### AND

(diagnosis OR “diagnostic accuracy” OR sensitivity OR specificity OR performance OR detection) No population terms or condition-specific filters (eg, BPH, dietary supplements, treatment outcomes).

The search will be conducted on PubMed (MEDLINE), CINHAL, Embase, Web of Science, Scopus, Cochrane Central Register of Controlled Trials and Google Scholar. No date restrictions will be applied, although we recognise that ULF-MRI is recent and most studies are post-2015. Searches will include all relevant studies published in English from database inception to February 2026. All search results will be imported into Mendeley Reference Manager. Duplicate citations will be identified and removed using the software’s de-duplication function prior to title and abstract screening.

### Other sources

This review will also include diagnostic accuracy studies, RCTs, non-randomised comparative studies (if there is not enough evidence from RCTs), cohort studies, case–control studies, cross-sectional studies, case series and case reports (even if they report relevant safety or effectiveness data), reviews and meta-analyses, editorials and commentaries, conference abstracts and in vitro (laboratory-based) studies.

### Data collection and analysis

#### Selection of studies

All studies that investigate the diagnostic accuracy of ULF-MRI for neurological imaging will be eligible for inclusion. Studies involving human participants of any age or sex will be included, with no restrictions based on health status or clinical indication.

Eligible studies will compare ULF-MRI for neurological imaging to one or more standard neurological imaging modalities, such as HF-MRI or CT. Comparators will be used to assess the relative accuracy of the imaging modality diagnosis.

### Data extraction and management

The retrieved data will be extracted from each included study, including the study characteristics (authors, publication year, country, study design and setting), participant/population details (sample size, age and referral symptoms/conditions), intervention details (ULF-MRI for neurological imaging, scanner field strength and imaging protocol), comparator details (standard neurological imaging (if applicable)) and imaging protocol applied. Primary and secondary outcomes include diagnostic accuracy metrics^23^ (sensitivity, specificity, accuracy/agreement, 2×2 contingency tables, PPV/NPV, SROC and AUC values), adverse events and level of evidence of each study and risk-of-bias assessments per study design. All data will be extracted independently by two reviewers (PL and EB) and managed using a standardised data extraction form (Microsoft Excel). Any discrepancies will be discussed and resolved by consensus. The systematic review was initiated in January 2026 and is expected to be completed by June 2026. The protocol was developed and finalised between January and February 2026. At the time of submission of this protocol manuscript, study identification, screening, data extraction, quality assessment and evidence synthesis have not yet commenced.

### Assessment of risk of bias in included studies

Risks of bias will be assessed using study design-specific tools for empirical (diagnostic accuracy) studies; this will include the Quality Assessment of Diagnostic Accuracy Studies 3 tool.^24^ If RCTs, observational studies, cohort studies, case–control studies, cross-sectional studies, case reports and systematic reviews are included, the Joanna Briggs Institute^25^ (JBI) critical appraisal checklist will be used. We recognise that empirical and non-empirical evidence are different levels of evidence. In this review, empirical studies will serve as the primary evidence base for the meta-analysis. Non-empirical sources, such as editorials, grey literature and narrative reviews, will be used to provide contextual information, identify knowledge gaps and support the discussion. Evidence will be reported separately by study type, and JBI appraisal tools will be applied as appropriate. The process will follow two reviewers independently assessing the literature; disagreements will be resolved through discussion. Risk-of-bias scores will be used to inform interpretation of results. No studies will be excluded solely based on quality rating.

### Measures of treatment effect

Diagnostic accuracy as categorical counts (frequencies) of agreement, sensitivity and specificity with corresponding 95% CIs will be applied to dichotomous data (frequency of events reported in 2×2 contingency tables). Pooled mean differences will be reported for data measured on identical scales and units applying meta-analysis through Meta-DiSc V.1.4.^26^ We evaluated Meta-DiSc V.2.0^27^ but retained Meta-DiSc V.1.4^26^ because it offered more comprehensive and detailed outputs for sensitivity and specificity forest plots, with numerical reporting of study-level proportions, 95% CIs, weights and heterogeneity statistics. During preliminary evaluation, the forest plots generated by Meta-DiSc V.2.0^27^ were less detailed for data extraction, transparency and reporting of raw data, and their SROC plots differed (compressed along the axis due to modelling) from conventional ROC presentations (curve plotting diagonally) traditionally used in published meta-analyses. Therefore, Meta-DiSc V.1.4^26^ was retained to ensure detailed, transparent and reproducible reporting and allow comparison of our results to other published studies.

### Dealing with missing data

For missing data in reporting studies, we will seek additional information from the study authors. If sufficient data cannot be obtained, they will be excluded from the data synthesis. Where feasible, the 2×2 contingency table analysis will be reported on the reported sample sizes and outcome measures to confirm results, including all participants in the groups to which they were originally assigned.

### Assessment of heterogeneity

The meta-analysis will use Meta-DiSc V.1.4 ^26^ to calculate the pooled estimates of sensitivity and specificity to assess diagnostic threshold heterogeneity inconsistency (*I*^2^). Further investigation will apply SROC using Moses’ constant of linear model (weighted regression and inverse variance) to calculate the accuracy of the results, with AUC values plotted.

### Assessment of reporting biases

Funnel plots will be employed to identify potential reporting biases and small-study effects. If the meta-analysis includes >10 studies, the Egger method^28^ will be applied to assess the source of any observed asymmetry.

### Data synthesis

All data will be synthesised with meta-analyses of forest plots, which will be conducted to assess pooled heterogeneity, p-values of the test and AUC. Tables will break down each study into categories of study design, sample size, imaging modality, outcome metrics and summary of findings.

### Subgroup analysis

Secondary outcomes will support the interpretation of the diagnostic accuracy of ULF-MRI for neurological imaging. These may include the lesion size and heterogeneity, sample size, reader demographics or size, lesion detection rate, image quality scores, reader confidence levels, reference standards used, arbitrator reviews, technical performance, environment of the imaging (community, laboratory, hospital, etc), MRI subcategories measured including imaging protocol, brain tissue measurements, subcortical grey/white matter structure volumes, etc. All outcomes will be reported descriptively.

### Sensitivity analysis

Results and effect estimate will include sensitivity, specificity, accuracy/agreement, 2×2 contingency tables, PPV/NPV, analysis of variance, ROC AUC, p-values, means, SD, risk ratios, ORs, CIs, etc.^23^

### Ethics and dissemination

Ethical approval is not required because the data used in this systematic review will be obtained from publicly available published studies and will not include individual personal^29^ or patient data,^30^ thereby eliminating privacy concerns. The results will be disseminated through publication in a peer-reviewed journal or presentation at a relevant academic conference.

## Discussion

The previous review^31^ was published 2 years ago; however, it did not include a meta-analysis; thus, an evidence gap remains. Additionally, the majority of the published studies have included a range of imaging protocols and study designs focused on brain volume^8 32^ (anatomical) measurements, or segmentation of anatomy,^33 34^ rather than the diagnosis of neurological pathology or disease, so it is necessary to assess the diagnostic efficacy of ULF-MRI for neurological imaging.

This systematic review has several limitations. Articles published in languages other than English will not be reviewed or analysed because of language barriers, which may introduce language bias. High heterogeneity may result from differences in sample sizes, lesion size and heterogeneity, neurological conditions imaged, and ULF-MRI protocols. Despite these limitations, this systematic review is expected to contribute to the current understanding of the diagnostic accuracy of ULF-MRI .

## Supplementary material

10.1136/bmjopen-2026-118511 online supplemental file 1
