# Supplementary material for: Diagnostic performance of ultra-low field neurological magnetic resonance imaging: a systematic review protocol
Source: BMJ Open. 2026 Sep 4;16(9):e118511. doi: 10.1136/bmjopen-2026-118511 (PMC13548277; doi:10.1136/bmjopen-2026-118511)
Supplement: Supplementary file 1 [file bmjopen-2026-118511.reviewer_comments.pdf]

# PEER REVIEW HISTORY

BMJ Open publishes all reviews undertaken for accepted manuscripts. Reviewers are asked to complete a checklist review form and are provided with free text boxes to elaborate on their assessment. These free text comments are reproduced below.

## ARTICLE DETAILS

### Title (Provisional)

The diagnostic performance of Ultra-Low Field neurological Magnetic Resonance Imaging: a systematic review protocol

### Authors

Lockwood, Paul; Barrett, Elizabeth

---

## VERSION 1 - REVIEW

---

| | |
|---|---|
| **Reviewer** | **1** |
| **Name** | **Mourato, Felipe** |
| **Affiliation** | **Brazilian Company of Hospital Services** |
| **Date** | **06-Apr-2026** |
| **COI** | **None** |

---

The manuscript describes the protocol for a systematic review and meta-analysis. Overall, the text is well-written and adequately covers all the necessary methodological domains for a protocol of this nature. However, I have a few methodological concerns that need to be addressed:

Analytical Software: The protocol mentions the use of Meta-Disc v1.4 software. While not inherently problematic, I question why more current tools, such as Meta-Disc 2.0, are not being utilized. Given that this is a "broad scope" systematic review—where authors will very likely need to perform diverse and complex analyses—the 2.0 version (Plana MN, Arevalo-Rodriguez I, Fernández-García S, et al. Meta-DiSc 2.0: a web application for meta-analysis of diagnostic test accuracy data. BMC Med Res Methodol. 2022;22(1):306. doi:10.1186/s12874-022-01788-2) would allow for more robust additional analyses. I strongly recommend updating the software choice or providing a clear justification for using the older version.

Inclusion Criteria vs. Quality Assessment: The authors state that in the absence of RCTs and diagnostic accuracy studies, they will include a broad range of study designs—such as editorials, previous reviews, and case series—using the Joanna Briggs Institute (JBI) critical appraisal tools. While JBI provides specific checklists for various study types, applying it across such a heterogeneous spectrum of evidence within a single review raises some

concerns. Attempting to synthesize and compare the risk of bias from empirical data (case series, for example) alongside non-empirical literature (expert opinions or editorials, for example) can lead to methodological problems. The authors must clarify how they intend to integrate such disparate levels of evidence in their final synthesis, as relying on JBI tools as a catch-all solution may mask intrinsic differences in evidence quality.

---


| | |
|---|---|
| **Reviewer** | **2** |
| **Name** | **Inati , Sara** |
| **Affiliation** | **National Institutes of Health, NINDS** |
| **Date** | **06-Jul-2026** |
| **COI** | **None** |


---

The aim of this systematic review protocol is to provide the current reported level of diagnostic accuracy for detecting intracranial abnormalities of ultra-low field magnetic resonance imaging (ULF-MRI) in pediatric and adult patients undergoing brain imaging for suspected neurological conditions/symptoms compared to conventional MRI and/or CT.  The literature was searched between February and March 2026.

I believe that this is a topic of considerable interest to the neuroimaging, radiologic and neurologic communities. However, the manuscript could be considerably improved through revision for grammar and clarity. It appears that the literature search has already been completed by March 2026.

Other comments:

Introduction: There was good discussion of the existing literature on technical efficacy of ULF-MRI for neurological imaging. There was less discussion of the extent of available studies on diagnostic accuracy in neurologic disorders and clear description of what has been done and what the exact gap is that this study will fill. Have enough clinical studies been performed to justify a meta-analysis? Some suggestion of what diseases have been studied to date would significantly improve the rationale for this meta-analysis.

Types of studies (and other sources): From what is written, it is unclear whether cohort studies, case-control studies, cross-sectional studies, etc. will be included or not included. Please clarify and justify.

Types of Interventions: Why is there no time frame for the included studies? It seems that this review should target studies using a certain set of technologies from a set timepoint (perhaps the earliest studies identified?)

It appears that non-English studies were excluded (mentioned in limitations). This should be noted as a criteria if true.

Types of outcome assessments: I am not sure why a primary outcome is "human participants as reader of imaging." Perhaps this should instead be listed as an inclusion or exclusion criteria.

Data extraction and management: how will this data be extracted and/or checked and how will discrepancies be resolved?

I am concerned that the overall metric of accuracy may be impacted by significant heterogeneity across lesion types. For example, I would a priori expect ULF-MRI to be better at detecting brain tumors vs subtle malformations of cortical development. I did not see this clearly listed in the subgroup analysis, even at the descriptive level, and I think this is an important clinical consideration as ULF-MRI may be more useful in some clinical situations than others. This is listed as a limitation, but I think needs to be accounted for in some way in the planned analyses in order for this study to have the impact that it seeks.

---

## VERSION 1 - AUTHOR RESPONSE

**Reviewer 1:** The manuscript describes the protocol for a systematic review and meta-analysis. Overall, the text is well-written and adequately covers all the necessary methodological domains for a protocol of this nature. However, I have a few methodological concerns that need to be addressed:

**Authors:** *Thank you for the peer review, and we appreciate the support provided by these comments in helping with our submission to the journal and disseminating this research to the wider international BMJ professional community.*

**Reviewer 1:** Analytical Software: The protocol mentions the use of Meta-Disc v1.4 software. While not inherently problematic, I question why more current tools, such as Meta-Disc 2.0, are not being utilized. Given that this is a "broad scope" systematic review—where authors will very likely need to perform diverse and complex analyses—the 2.0 version (Plana MN, Arevalo-Rodriguez I, Fernández-García S, et al. Meta-DiSc 2.0: a web application for meta-analysis of diagnostic test accuracy data. BMC Med Res Methodol. 2022;22(1):306. doi:10.1186/s12874-022-01788-2) would allow for more robust additional analyses. I strongly recommend updating the software choice or providing a clear justification for using the older version.

**Authors:** *We thank the reviewer for this insightful comment and for highlighting Meta-DiSc v2.0. We carefully evaluated both Meta-DiSc v1.4 and Meta-DiSc v2.0 during protocol development. Our decision to use Meta-DiSc v1.4 was based on several practical and methodological considerations relevant to the planned analyses. Specifically, Meta-DiSc v1.4 provides detailed conventional forest plot outputs for meta-analysis (sensitivity and specificity estimates), specifically Meta DiSc v.1.4 provides detailed numerical data reporting within the outputted plots (the proportions with 95% confidence intervals, study weights, heterogeneity statistics, pooled estimates, and other numerical parameters) that can be directly reported for transparency and rigor of the meta-analysis for the reader to review on the forest plot output. In contrast, while Meta-DiSc v2.0 offers a more modern web-based interface, we found its graphical forest plot outputs (particularly the sensitivity and specificity forest plots) provide none of the detailed numerical information (numerical data reporting the proportions with 95%*

*confidence intervals, study weights, heterogeneity statistics, and other numerical parameters) for reporting purposes; these less detailed outputs were less informative for the reader. Furthermore, during our preliminary assessment, the SROC plots generated by Meta-DiSc v2.0 (bivariate random-effects model) appeared visually different from conventional ROC representations, with the curve tending to be graphically asymmetric and shifted toward the sensitivity axis (upper left compressed to the axis as consequence of the modelling used by Meta-DiSc v2.0), which made interpretation and comparison with previously published DTA ROC meta-analyses more challenging. Whereas the Meta-DiSc v1.4 produces traditional ROC curve plotting graphically (Moses-Littenberg SROC approach) going from the usual 0-100, diagonally curved across the plot as is normal for ROC presentations, which the authors felt was a reasonable and practical justification for its use due to these traditional reporting and presentation styles that allow our data to be compared to other meta-analysis publication outputs.*

*As our primary objective was to conduct a transparent and reproducible meta-analysis using established and widely cited methodology, Meta-DiSc v1.4 was considered the most suitable tool for generating the detailed graphical outputs (forest plots with numerical data and SROC) required for our analyses and reporting. Nevertheless, we acknowledge Meta-DiSc v2.0 has methodological differences and some statistical changes, but we acknowledge it isn't always the best choice for every review; such as this instance, but we will consider its use again in the future, where forest plots and SROC may not warrant such detailed visualisation of numerical data transparency for the reader. This justification has been added to the protocol to clarify the rationale for our software choice.*

**Reviewer 1:** Inclusion Criteria vs. Quality Assessment: The authors state that in the absence of RCTs and diagnostic accuracy studies, they will include a broad range of study designs—such as editorials, previous reviews, and case series—using the Joanna Briggs Institute (JBI) critical appraisal tools. While JBI provides specific checklists for various study types, applying it across such a heterogeneous spectrum of evidence within a single review raises some concerns. Attempting to synthesize and compare the risk of bias from empirical data (case series, for example) alongside non-empirical literature (expert opinions or editorials, for example) can lead to methodological problems. The authors must clarify how they intend to integrate such disparate levels of evidence in their final synthesis, as relying on JBI tools as a catch-all solution may mask intrinsic differences in evidence quality.

**Authors:** *We thank the reviewer for this important observation and agree that combining empirical and non-empirical sources of evidence without clear distinction could introduce methodological challenges. The intent of our broad inclusion criteria is to ensure a comprehensive assessment of the available literature in an area where high-level evidence (e.g., RCTs and diagnostic accuracy studies) may be limited. However, we do not intend to synthesise empirical and non-empirical evidence at the same evidentiary level. Specifically, empirical studies (e.g., diagnostic accuracy studies, cohort studies, case-control studies, and other primary empirical research designs) will form the basis of the primary evidence synthesis and meta-analysis. These studies will undergo design-specific critical appraisal using the relevant QUADAS-3 or relevant JBI tool to assess their methodological characteristics and risk of bias.*

*In contrast, non-empirical publications such as editorials, expert opinions, commentaries, and narrative reviews will not be included in the meta-analysis. Rather, these sources will be used to*

*provide contextual information, identify knowledge gaps, describe emerging concepts, and assist in interpreting findings within the discussion section.*

*We have revised the protocol to clarify that inclusion of these sources is intended to inform the broader interpretation of the evidence base rather than contribute directly to estimates of effect or diagnostic performance.*

*We agree that JBI critical appraisal tools should not be viewed as a mechanism for equating different levels of evidence. Instead, they will be applied using the study-design-specific checklists recommended by JBI, with the resulting assessments interpreted within the context of each evidence type. This clarification has been added to the protocol.*

**Authors:** *Again, we thank you for your time and comments and hope the revisions will now be accepted for publication.*

------------------------------------------------------------------------------------------------------------

**Reviewer 2:** The aim of this systematic review protocol is to provide the current reported level of diagnostic accuracy for detecting intracranial abnormalities of ultra-low field magnetic resonance imaging (ULF-MRI) in pediatric and adult patients undergoing brain imaging for suspected neurological conditions/symptoms compared to conventional MRI and/or CT. The literature was searched between February and March 2026.

**Authors:** *Thank you for the peer review, and we appreciate the support provided by these comments in helping with our submission to the journal and disseminating this research to the wider international BMJ professional community.*

**Reviewer 2:** I believe that this is a topic of considerable interest to the neuroimaging, radiologic and neurologic communities. However, the manuscript could be considerably improved through revision for grammar and clarity. It appears that the literature search has already been completed by March 2026.

**Authors:** *Thank you, we have revised the protocol manuscript to improve grammar, readability, and clarity throughout. With regard to the literature search, the reviewer is correct that the search had already been completed by March 2026. The final systematic literature article has since been submitted to BMJ and is currently going through peer-review.*

**Reviewer 2:** Other comments: Introduction: There was good discussion of the existing literature on technical efficacy of ULF-MRI for neurological imaging. There was less discussion of the extent of available studies on diagnostic accuracy in neurologic disorders and clear description of what has been done and what the exact gap is that this study will fill. Have enough clinical studies been performed to justify a meta-analysis? Some suggestion of what diseases have been studied to date would significantly improve the rationale for this meta-analysis.

**Authors:** *We thank the reviewer for this valuable comment; there are a few items raised, and we shall endeavour to unpick and answer each.*

*We agree that the protocol does not imply it already knows the extent of the current clinical evidence base for ultra-low-field MRI (ULF-MRI) in neurological disorders; the search method in*

*the protocol instead details for the reader how it will identify the number of published studies and then assess them.*

*Although the Introduction does summarise the technical feasibility and image quality literature, we acknowledge that it provides less detail on the emerging body of evidence evaluating diagnostic performance of ULF-MRI in specific neurological conditions; the aims in the protocol instead details for the reader that is the research question to find the number of published studies and then assess them.*

*Likewise, the protocol at the time of writing had not completed the literature search to answer your question of* 'have enough clinical studies been performed to justify a meta-analysis' *it is the aim of the protocol to answer this question of whether sufficient studies exist to justify a meta-analysis. This protocol has been designed prospectively to identify and evaluate the available evidence. Meta-analysis will only be undertaken where sufficient comparable data are available; otherwise, findings will be synthesised narratively in accordance with established systematic review methodology. This approach ensures that the review remains valid regardless of the eventual volume or heterogeneity of the evidence identified.*

*We have therefore revised the Introduction as asked to include a brief overview of the neurological applications that have been investigated to date, including areas such as stroke, hydrocephalus, intracranial pathology, multiple sclerosis, neurodegenerative disorders, and other neurological conditions where ULF-MRI has been applied.*

*We have also expanded the discussion section on the current evidence gap. While several studies have reported diagnostic findings in clinical populations, the literature remains fragmented across different neurological disorders, imaging protocols, and study designs. To our knowledge, no systematic review and meta-analysis has synthesised the available diagnostic accuracy evidence across these applications.*

**Reviewer 2:** Types of studies (and other sources): From what is written, it is unclear whether cohort studies, case-control studies, cross-sectional studies, etc. will be included or not included. Please clarify and justify.

**Authors:** *Thank you. Under the 'types of studies' subheading, we have listed all relevant types of studies that would be included if they contain diagnostic accuracy of ULF-MRI, and this does, as you say, include all of the above.*

**Reviewer 2:** Types of Interventions: Why is there no time frame for the included studies? It seems that this review should target studies using a certain set of technologies from a set timepoint (perhaps the earliest studies identified?)

**Authors:** *Thank you, under the 'electronic searches' section we have updated the wording to include "No date restrictions will be applied, although we recognise that ULF-MRI is recent and most studies are post-2015".*

**Reviewer 2:** It appears that non-English studies were excluded (mentioned in limitations). This should be noted as a criteria if true.

**Authors:** *Thank you, this has now been amended as advised.*

**Reviewer 2:** Types of outcome assessments: I am not sure why a primary outcome is "human participants as reader of imaging." Perhaps this should instead be listed as an inclusion or exclusion criteria.

**Authors:** *Thank you, the exclusion criteria does clarify "Artificial Intelligence (AI) tools as the reader/interpretation", and as you say, the types of primary outcomes of studies include "human participants as readers of imaging (most commonly a consultant neuroradiologist but maybe other related healthcare professions). This study will not be using AI as a reader". We wanted to underline this in both sections to highlight that this is not a study assessing AI tools.*

**Reviewer 2:** Data extraction and management: how will this data be extracted and/or checked and how will discrepancies be resolved?

**Authors:** *Thank you, although we had discussed the process would involve two reviewers independently assessing the risk of bias, the data extraction of who had been omitted from the 'data extraction' section due to word count; this, as advised, has now been amended to include this important fact.*

**Reviewer 2:** I am concerned that the overall metric of accuracy may be impacted by significant heterogeneity across lesion types. For example, I would a priori expect ULF-MRI to be better at detecting brain tumors vs subtle malformations of cortical development. I did not see this clearly listed in the subgroup analysis, even at the descriptive level, and I think this is an important clinical consideration as ULF-MRI may be more useful in some clinical situations than others. This is listed as a limitation, but I think needs to be accounted for in some way in the planned analyses in order for this study to have the impact that it seeks.

**Authors:** *Thank you for highlighting this, and indeed it is, as you say, listed in the very final paragraph as a limitation to the reader. We agree that diagnostic performance may and most likely (a priori) will vary substantially across lesion types, and that ULF-MRI may demonstrate greater accuracy for some pathologies than others. However, as this is a research protocol, lesion-specific data was unknown at time of protocol submission to the journal, and the research aim is to assess baseline performance in detecting various neurological conditions.*

*We understand your concern and acknowledge this expectation as reasonable, to address this issue, we have added it as an additional subgroup analyses (lesion size and heterogeneity). We agree that understanding whether ULF-MRI performs better in certain clinical contexts is highly relevant and will be explored wherever the available evidence permits.*

**Authors:** *Again, we thank you for your time and comments and hope the revisions will now be accepted for publication.*

---

## VERSION 2 - REVIEW

---

| | |
|---|---|
| **Reviewer** | **2** |
| **Name** | **Inati , Sara** |

| | |
|---|---|
| **Affiliation** | **National Institutes of Health, NINDS** |
| **Date** | **07-Aug-2026** |
| **COI** | |

My comments have been adequately addressed.
