# Supplementary material for: Diagnostic performance of ultra-low field neurological magnetic resonance imaging: a systematic review protocol
Source: BMJ Open. 2026 Sep 4;16(9):e118511. doi: 10.1136/bmjopen-2026-118511 (PMC13548277; doi:10.1136/bmjopen-2026-118511)
Supplement: Supplementary file 2 [file bmjopen-16-9-s001.docx]

**Title:** The diagnostic performance of Ultra-Low Field neurological Magnetic Resonance Imaging: a systematic review protocol

**Authors:**

Paul Lockwood

School of Nursing, Midwifery, Allied and Public Health, Canterbury Christ Church University, Kent, United Kingdom

Elizabeth Barrett

Kent and Medway Medical School, Canterbury Christ Church University and University of Kent, Kent, United Kingdom.

Radiology Department, Darent Valley Hospital, Dartford and Gravesham NHS Trust, Kent, United Kingdom

**Correspondence:** Paul Lockwood paul.lockwood@canterbury.ac.uk
